# Bacterial isolation and genome analysis of a novel *Klebsiella quasipneumoniae* phage in southwest China’s karst area

**DOI:** 10.1186/s12985-024-02321-1

**Published:** 2024-03-06

**Authors:** Yanju Liu, Jinfeng Wang, Ruoyu Zhao, Xiaoping Liu, Yang Dong, Wenyu Shi, Hongchen Jiang, Xiangyu Guan

**Affiliations:** 1 School of Ocean Sciences, China University of Geosciences (Beijing), No. 29 Xueyuan Road, Haidian District, 100083 Beijing, China; 2 College of Food Science & Nutritional Engineering, China Agricultural University, 100083 Beijing, China

**Keywords:** Karst, Bacterial isolation, *Klebsiella* phage, *Autophagoviridae*, Genome

## Abstract

**Background:**

Southwest China is one of the largest karst regions in the world. Karst environment is relatively fragile and vulnerable to human activities. Due to the discharge of sewage and domestic garbage, the karst system may be polluted by pathogenic bacteria. The detection of bacterial distribution and identification of phage capable of infecting them is an important approach for environmental assessment and resource acquisition.

**Methods:**

Bacteria and phages were isolated from karst water in southwest China using the plate scribing and double plate method, respectively. Isolated phage was defined by transmission electron microscopy, one-step growth curve and optimal multiplicity of infection (MOI). Genomic sequencing, phylogenetic analysis, comparative genomic and proteomic analysis were performed.

**Results:**

A *Klebsiella quasipneumoniae* phage was isolated from 32 isolates and named KL01. KL01 is morphologically identified as *Caudoviricetes* with an optimal MOI of 0.1, an incubation period of 10 min, and a lysis period of 60 min. The genome length of KL01 is about 45 kb, the GC content is 42.5%, and it contains 59 open reading frames. The highest average nucleotide similarity between KL01 and a known *Klebsiella* phage 6939 was 83.04%.

**Conclusions:**

KL01 is a novel phage, belonging to the *Autophagoviridae*, which has strong lytic ability. This study indicates that there were not only some potential potentially pathogenic bacteria in the karst environment, but also phage resources for exploration and application.

**Supplementary Information:**

The online version contains supplementary material available at 10.1186/s12985-024-02321-1.

## Introduction

Southwest China is one of the largest karst zones in the world [1]. The total natural resources of karst groundwater is 2039.67 × 10^8^ m^3^/a, accounting for 23.4% of the total groundwater in China [2, 3]. In southwest China, karst water is indispensable for local agriculture, industry, and public drinking water supply [4–6]. The characteristic of karst groundwater is high conductivity, high flow rate and rapid infiltration. As a result, karst groundwater was easily contaminated by human activities such as industry, urbanization and agriculture, which would threaten the water supply security in this area [7, 8].

Microbial contamination is a persistent water quality problem worldwide and poses a significant risk to public health [1, 9, 10]. Meanwhile, the existence of antibiotic resistant bacteria would cause the multiple antibiotic resistant genes (ARGs) transmitted in karst river would further increase the risk to human health [11]. It was monitored that bacterial pathogens including verotoxin-producing *Escherichia coli* (*E. coli*), *Campylobacter spp.* and *Salmonella spp*., as well as *Pseudomonas aeruginosa* were distributed in karst ecosystem [12]. Conditional pathogenic bacteria in karst water system, such as *Bacteroides* and *Aureobacterium*, were found and the detection rate is different from the upstream and downstream of karst river [13]. Due to the spatial difference and hydrological complexity of karst systems, the understanding of microbes in karst systems is still limited.

To the bacterial pathogens, bacteriophages are their natural enemies [14, 15]. Owing to the ubiquity and abundance of phages, they are responsible for 20–40% of bacterial lysis events, and are widely used in environmental pathogen control [16, 17]. Bacteriophages in natural water can prevent the migration of pathogens from soil to groundwater, reduce the abundance of pathogens, and decrease the migration risk of pathogens [18]. Lytic phage could reduce the proportion of the isolated antibiotic-resistant bacteria (mainly *Aeromonas* species) from 65.7 to 20% in 24 h from wastewater treatment plants (WWTPs), and control the soil-borne pathogen *Ralstonia solanacearum* [19, 20]. It follows that phages represent a force in shaping the composition of the bacterial pathogens in environments. Therefore, it was valuable to further identify and then exploit well-known and novel phage resources for applications in environment. Meanwhile, it was also important to understand the biology of bacteriophages, such as their host specificity, genomic diversity, and host adaptation.

In this study, a typical karst river in southwest China was studied. The study area is close to sewage treatment plants and residential areas, and there may be the possibility of microbial exchange between the study area and the surrounding environment. We investigated the bacteria living in the studied sites, searched for potential pathogenic bacteria and isolated phage against certain bacteria. The morphology, host range, and growth parameters of phage was characterized. Genomic sequencing, phylogenetic analysis, comparative genomic and proteomic analysis were performed. The research results can indicate whether there are potential pathogenic bacteria and biological risks in karst area, and rich phage species resources.

## Methods

### Site description and sampling

There are two karst river systems in the study area, located in Kaiyang County, Guizhou Province, China. In this area, there are no large-scale livestock farms in the study area, but there are WWTPs with serious trash dumping conditions by residents around the lower course of river. We collected samples in August 2017, with a total of five sampling locations, each having three replicates (Table 1).

**Table 1:** Site description and sampling

Sampling Site	Sampling Time	Sampling Description	Longitude	Latitude	Bacteria Isolation	Phage Isolation
KY12	2017.8	Karst surface water	106°59′31.46″	27°03′12.95″	8	1
KYX03	2017.8	Karst surface water	106°59′10.07″	27°03′44.30″	2	0
TK01	2017.8	Karst groundwater	107°00′41.93″	27°04′21.92″	11	0
WWTP	2017.8	Sewage treatment plant intake	107°01′6.67″	27°04′54.21″	7	0
WWTP2	2017.8	Sewage treatment plant outlet	106°59′12.7″	27°05′6.82″	4	0

### Bacterial culture and isolation

Firstly, the collected water samples were filtered through a 0.22 μm filter membrane to enrich microorganisms. Then 5 g of sediment sample and filter membrane were placed into 100 mL Brian Heart Infusion (BHI) (1,000 mL purified water, 17.5 g brain and heart infusion powder, 2.0 g D-glucose, 10.0 g peptone, 5.0 g NaCl, 2.5 g Na_2_HPO_4_, pH = 7.4) to enrichment culture at 37℃ [21]. After the serial dilutions of enriched culture liquid, diluted samples added to the BHI culture plate and spread. The petri-plates were then incubated in an inverted position for 24–72 h at 37℃. Single colonies were picked up according to different shapes and colors of the colonies on the plate, and single colonies were purified by streak plate method. Repeat the previous step three times until the characteristics of the colonies in the plate are consistent.

### 16S rRNA gene sequencing and identification

PCR amplification and sequencing were performed to confirm the identity of the bacteria isolate using universal primers (27 F’- AGAGTTTGATCCTGGCTCAG and 1492 R’- TACGGCTACCTTGTTACGACTT for the 16S rRNA gene) [22]. The PCR product was run on a 1.5% (w/v) agarose gel to identify its size. The purified PCR products were sent to Beijing Rubo Xinke Company (China) for 16S rRNA gene sequencing, and Blast homology analysis was conducted on the NCBI website (NCBI; http://blast.ncbi.nlm.nih.gov). The phylogenetic analysis was performed based on 16S rRNA gene sequences and established the phylogenetic position of the potentially pathogenic microorganisms aligned with the MUSCLE alignment through the Neighbor-Joining method by the most closely related bacterial type strains [23]. The phylogenetic tree was constructed using MEGA X software [24].

### Bacterial resistance detection

The Kirby-Bauer disk diffusion method was applied to determine the antibiotic susceptibility pattern [25]. The results were interpreted according to guidelines from the European Committee on Antimicrobial Susceptibility Testing (EUCAST) [26]. The antibiotics for antibiotic susceptibility test were selected based on the concentration and types of common antibiotics in the actual environment. Finally, five antibiotics were selected, including ofloxacin, sulfadiazine, tetracycline, lincomycin, and azithromycin. Each antibiotic was set with five qualities (0.5 µg, 1 µg, 5 µg, 10 and 15 µg). A sterilized cotton swab was dipped in the bacterial liquid (0.5 Mclntyre turbidimetric tube) to evenly smear it on the surface of the MH solid plate. Thereafter, 6 mm discs with discrete doses of antibiotics were applied onto the MHA plate surfaces and incubated at 37 °C for 24 h. Three parallel groups were set for each experiment and measured the inhibition zones diameter.

### Phage isolation

Briefly, 100 mL water samples centrifuged at 12,000 rpm for 3 min to remove large impurities. Then filtered the supernatant through 0.45 and 0.22 μm filter membrane to remove bacteria. The filtered water was then added to BHI with potential pathogenic bacteria cultured and incubated at 37 °C for 24 h to enrich for phages. The enrichment culture was centrifuged and filtered with a 0.22 μm filter membrane to remove cellular debris. Then, the phage enriched filtrate was serially diluted in supermicro buffer (SM buffer) (5.8 g NaCl, 2 g MgSO_4_·7H_2_O, 50 mL 1 mol/L Tris-HCl, 5 mL 2% gelatin, add distilled water to 1 L, pH = 7.5) and used in double agar method to carry out the plaque assays, 100 µL of phage enriched filtrate was mixed with 100 µL logarithmic phase bacteria for 15 min. To this we added 3 mL BHI (0.75% Agar) and poured on the plate containing BHI (1.5% Agar) [27, 28]. After overnight incubation, a single plaque was collected for further purification by replating three times until the uniform size, shape, and clarity of plaques were obtained.

### Morphological identification of phage

Morphological features of the isolated bacteriophage (KL01) were observed by transmission electron microscopy (TEM) [29, 30]. A high concentration of phage KL01 solution was prepared for negative staining to analyze the morphology. The 10 mL supernatant was added to a 20% polyethylene glycol 8,000 solution containing 2.5 M NaCl and then incubated for 1 h on ice. The phage pellets were collected by centrifugation at 10,000×g at 4 °C for 15 min and then resuspend in a small volume of SM buffer (150 µL) to obtain a high concentration of phage solution [31–33]. Firstly, phage particles were adsorbed on elastic carbon-support film copper grids (Okenshoji Co., Ltd., Tokyo, Japan) for 5 min, and the excess liquid was removed using a filter paper (Advantec Toyo Kaisha, Ltd., Tokyo, Japan). Secondly, phage particles were negatively stained with adding 10 µL 2% phosphotungstic acid. The morphological characteristics of phages were observed with TEM after natural drying (JEM-1200EX, Japan). It was classified and identified based on International Committee on Taxonomy of Viruses (ICTV) guidelines.

### Optimal MOI determination

The multiplicity of infection (MOI) determination was carried out as the reference research [34–36]. Phage KL01 was incubated with the log-phase culture of *K. quasipneumoniae* (10^8^ CFU/mL) at various proportions (10, 1, 0.1, 0.01, 0.001 and 0.0001) followed by incubation at 37 °C 170 rpm for 5 h. The titers of the phage at different MOI were determined by the double-agar layer plate method. Each experiment was set up with three parallel groups, and the optimal MOI of KL01 was finally obtained.

### One-step growth and adsorption curve determination

The one-step growth curve was generated according to a previous report with minor modifications [37]. According to the optimal MOI 0.1, phage KL01 (~1 × 10^7^ PFU/mL)was incubated with exponential phage *K. quasipneumoniae* (~1 × 10^8^ CFU/mL) [35, 36]. After incubated at 37 °C for 10 min, the mixture was centrifuged at 12,000 rpm for 5 min at 4 °C to remove the unabsorbed phage in the supernatant by repeated three times. The centrifuged precipitation was then resuspended in BHI broth followed by incubation at 37 °C 170 rpm. The total sampling time was 180 min to determine the phage titer, with samples taken every 5 min in the first 30 min and every 10 min in the remaining 150 min. Three parallel groups were set for each experiment. The phage adsorption experiment according to the optimal MOI, bacteria (~1 × 10^8^ CFU/mL) in the logarithmic growth stage and phage (~1 × 10^7^ PFU/mL) were added, incubated for 20 min, sampled for centrifugation and filtration at an interval of 2 min to remove the host bacteria [38]. The concentration in supernatant was determined by double-layer flat plate method, and three parallel experiments were set.

### Host range analysis

The range of bacteriophage lysis spectrum was determined by standard spot tests assay [39]. The spot assay was carried out on the phage KL01 to determine the host specificities, and plaque assays were performed on 32 isolated bacteria. The 5 µL of each phage suspension was placed on each lawn plate of candidates. The plates were then incubated at 37 °C and examined for plaques after 3–24 h. Bacterial sensitivity to KL01 was established by a clear lysis zone at the spot. Based on the clarity of the spot, bacteria were divided into two categories: clear lysis zone (+) and no lysis zone (−). Three parallel groups were set in each experiment, and the culture dish without adding KL01 was used as the blank control group.

### Extraction of genomic DNA from phage

Phage DNA was extracted using λ phage genomic DNA rapid extraction kit (Beijing Adlai Company, China) according to the manufacturer’s instructions. The extracted DNA was fragmented to construct libraries with 300 bp inserted length, and the 150 bp paired-end sequencing was performed on the Illumina X-ten platform (Shanghai Majorbio Technology, Shanghai, China).

### Genome analysis

Sickle (v 1.33) was used for quality control to remove sequencing adapters and low-quality reads [40]. The default parameters of SPAdes (v3.13.0, -- meta - k 17, 31, 51, 71, 91) were used to splice and assemble high-quality reads [41]. The completeness and contamination of the obtained contigs were evaluated using check M (v1.0.13) [42]. For potentially low abundance contaminated sequences, the Expectation Maximization (EM) algorithm was used to split the joint multimodal distribution of GC content and coverage, extracting high abundant bacteria and phages, and then using check M to evaluate their completeness and contamination. Bowtie2 was used to map the sequencing reads to both ends of the phage sequence, detecting the consistency of the abundance and average coverage of reads across the head and tail of the KL01 genome sequence, and further verifying the completeness of the genome [43].

VectorBuilder (https://www.vectorbuilder.cn/tool/gc-content-calculator.html) was used for the composition analysis of the KL01 genome, including genome size, GC content, and base composition. Potential ORFs of the KL01 genome were predicted using combined server from GeneMarkS (http://exon.gatech.edu/) [44] and Rapid Annotation using Subsystem Technology (RAST). Protein function was predicted using the NR database of the BLASTp and annotated functional genes were manually corrected after searching domains on the SMART (http://smart.embl-heidelberg.de/). The presence of tRNA was determined using tRNAscan-SE (http://trna.ucsc.edu/tRNAscan-SE/). Searching of antibiotic resistance genes were carried out using database ARDB (http://ardb.cbcb.umd.edu/) and integrative-comprehensive database of virulence factors (VFDB, http://www.mgc.ac.cn/VFs/). The genome map, GC distribution, offset, and protein structure of bacteriophages were visualized through the online website CGview Server [45].

The average nucleotide identity (ANI) was determined among all pairwise combinations of phage genomes, using the BLASTn alignment tool in the pyani package and plotted in an interactive heatmap to identify phages [46]. Phylogenetic trees were based on neighbor-joining methods and constructed using MEGA X [24]. Bootstrap resampling was performed for 1,000 replications and the sequences were aligned using Clustal W [47]. Mauve 2.3.1 [48] was used to compare and analyze multiple phage genomes, calculate the homology among genome sequences, and generate the visualization of genome comparison.

The amino acid sequences of the lysozyme and holin of KL01 were entered into ProtParam (https://web.expasy.org/protparam/) to analyze the physicochemical properties of the proteins. Then used SignalP-6.0 (https://services.healthtech.dtu.dk/service.php?SignalP) and TMHMM-2.0 (https://services.healthtech.dtu.dk/service.php?TMHMM-2.0) for predicting the protein signal peptide and transmembrane structural domain.

### Proteomics analysis

Cultured phage KL01 supernatants were sent to Shanghai Majorbio Technology for proteomic analysis. The protein concentration was determined by BCA (bicinchoninic acid) method, and the protein quality was determined by SDS-PAGE (sodium dodecyl sulfate-polyacrylamide gel). Reductive alkylation and trypsin digestion were carried out for the protein samples with acceptable quality. Finally, liquid chromatography coupled with tandem mass spectrometry (LC-MS/MS) analysis was performed. For the protein database search comparison, the results obtained by data statistics and bioinformatics analysis.

## Results

### Isolation and identification of potential pathogenic bacteria

**Fig. 1:**
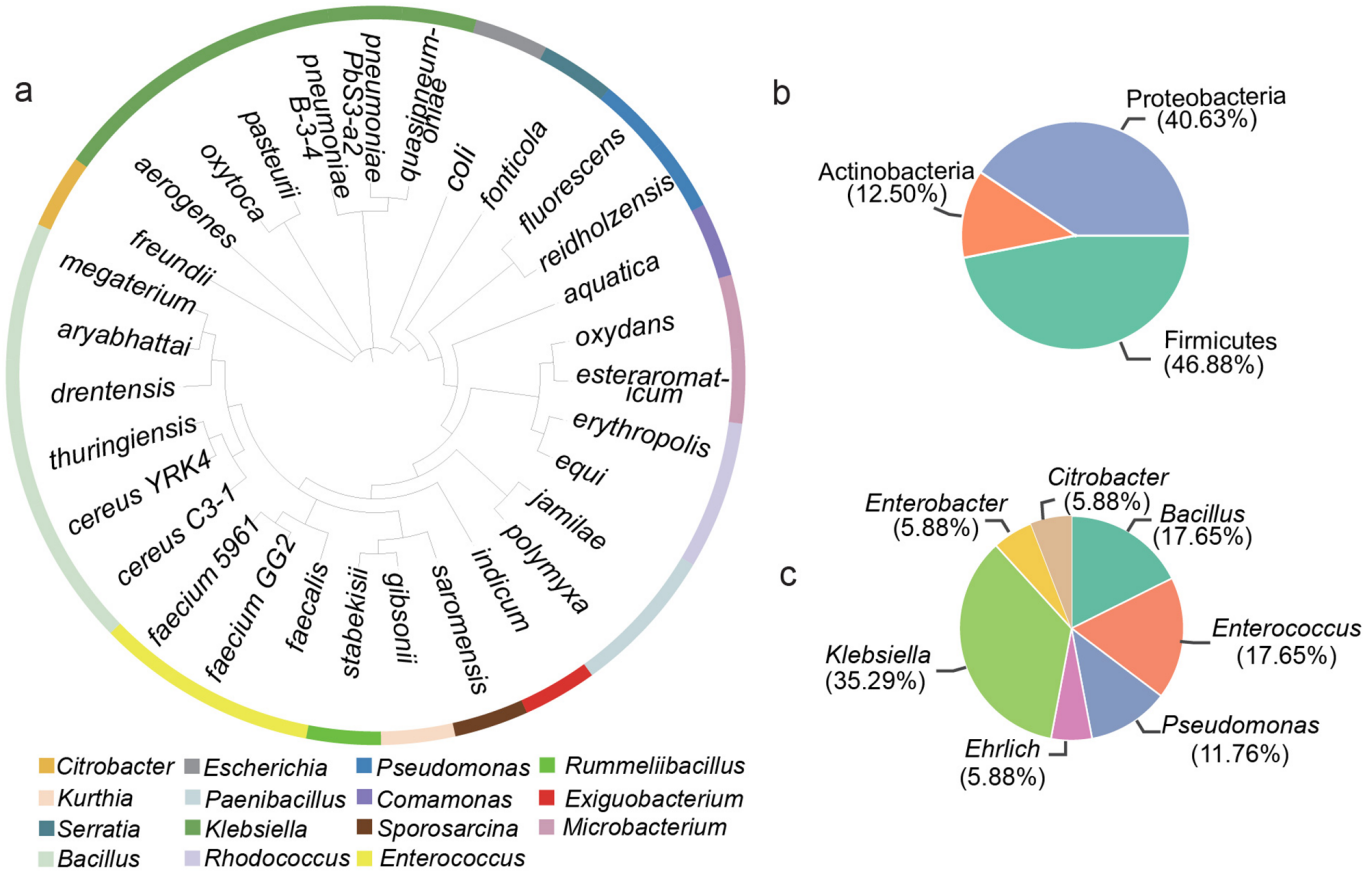
Classification and prevalence of bacteria isolated from the study area. **a.** 32 isolates were identified based on 16S rRNA gene sequencing. Each color block represented a different genus. **b**. The proportion of isolated bacteria at phylum level. **c**. The distribution of reported potential pathogenic bacteria in the isolated bacteria.

We isolated 32 bacteria from the collected samples through microbial isolation and cultivation (Table 1), and identified them based on 16S rRNA gene sequence (GenBank ID: PP178668-PP178699), which were classified into 15 genera (Fig. 1a). These genera are classified into three phyla, including Proteobacteria, Firmicutes and Actinobacteria, accounting for 40.63%, 46.88% and 12.50%, respectively (Fig. 1b). 17 of the isolated genera have been reported to have potential pathogenicity, including *Klebsiella*, *Enterococcus* and *Bacillus* (Fig. 1c). Meanwhile, *Pseudomonas* and *E. coli* were isolated, both of which belong to conditional pathogens.

### Phage isolation and its biological properties

**Fig. 2:**
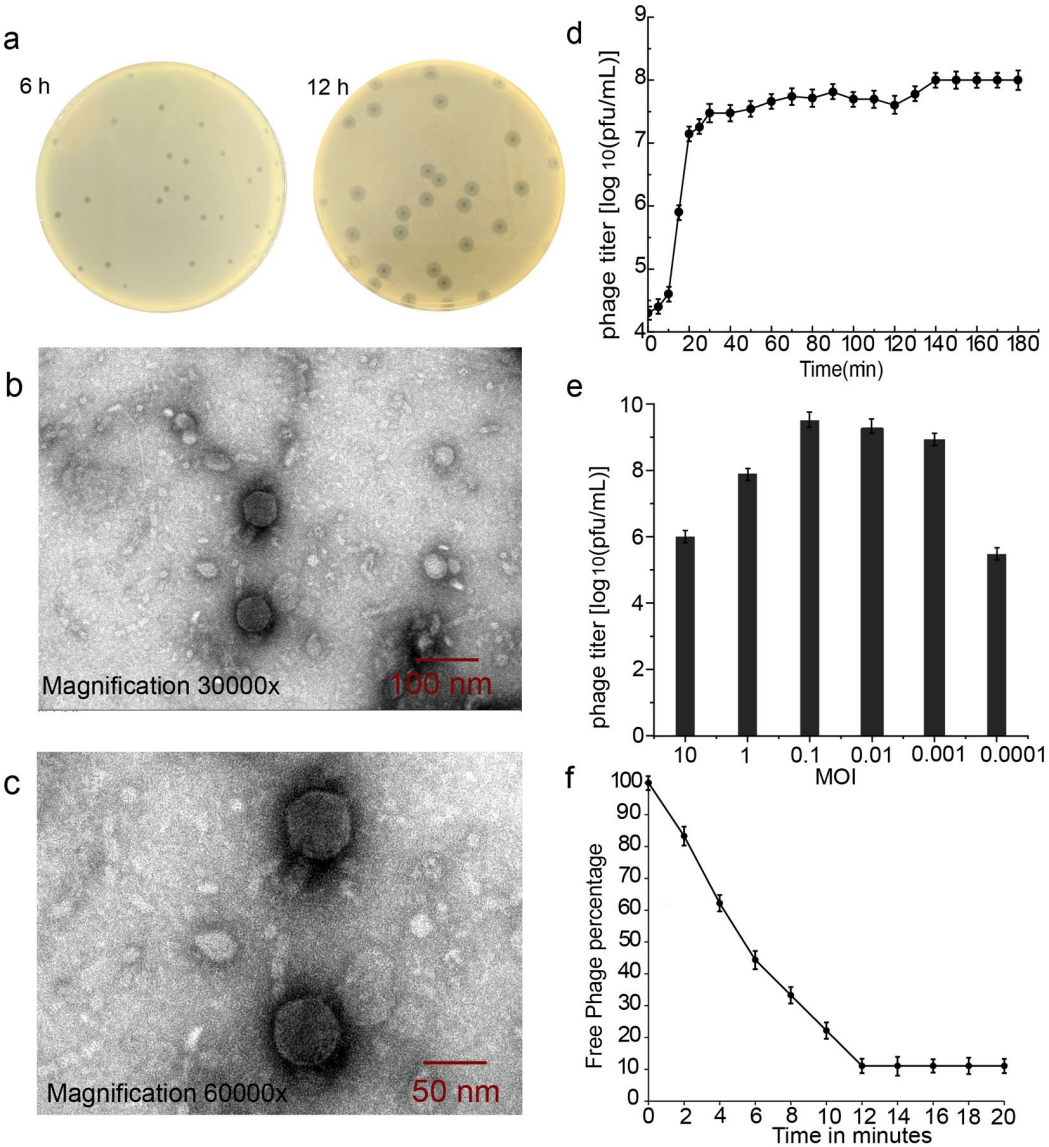
Basic biological characteristics of *Klebsiella quasipneumoniae* phage KL01. **a**. The plaque of phage KL01. **b-c**. Transmission electron microscope image of phage KL01. **d**. The one-step growth curve of phage KL01. **e**. The optimal MOI of KL01. **f**. The adsorption curve of phage KL01.

The 32 isolates were used for host phage isolation, but only the bacteriophage of *K. quasipneumoniae* was isolated and named KL01. The KL01 titer was determined to be 1 × 10^8^ PFU/mL. The purified plaque has halo zone, uniform morphology and size, clear edges, and a diameter of about 2 mm as shown in Fig. 2a. The transmission electron microscopy results revealed KL01 has a head and tail structure, and the head was symmetric, and diameter was approximately 60 nm (Fig. 2b and c). It belongs to the *Caudoviricetes*.

The titer of phage KL01 reached its maximum value (3.2 × 10^9^ PFU/mL) when the MOI was 0.1, and the propagation of their progeny is the greatest (Fig. 2d). The one step growth and adsorption curve showed that the latency period of KL01was approximately 10 min and the lysis period was 60 min (Fig. 2e and f), which indicated that KL01 had a high lysis efficiency and could rapidly infect the host bacteria *K. quasipneumoniae*. KL01 infected with only one of 32 strains of bacteria and could not infect *Klebsiella oxytoca* and other bacteria in the same environment (Table S1). According to the EUCAST, the host bacteria *K. quasipneumoniae* were resistant to sulfadiazine, lincomycin, azithromycin, and ofloxacin, and only ofloxacin had an inhibition zone with a diameter of less than 20 mm. The bacteria were not resistant to tetracycline and its inhibitory zone was directly 18 mm (Fig. S1).

### Genomic characterization of phage KL01

**Fig. 3:**
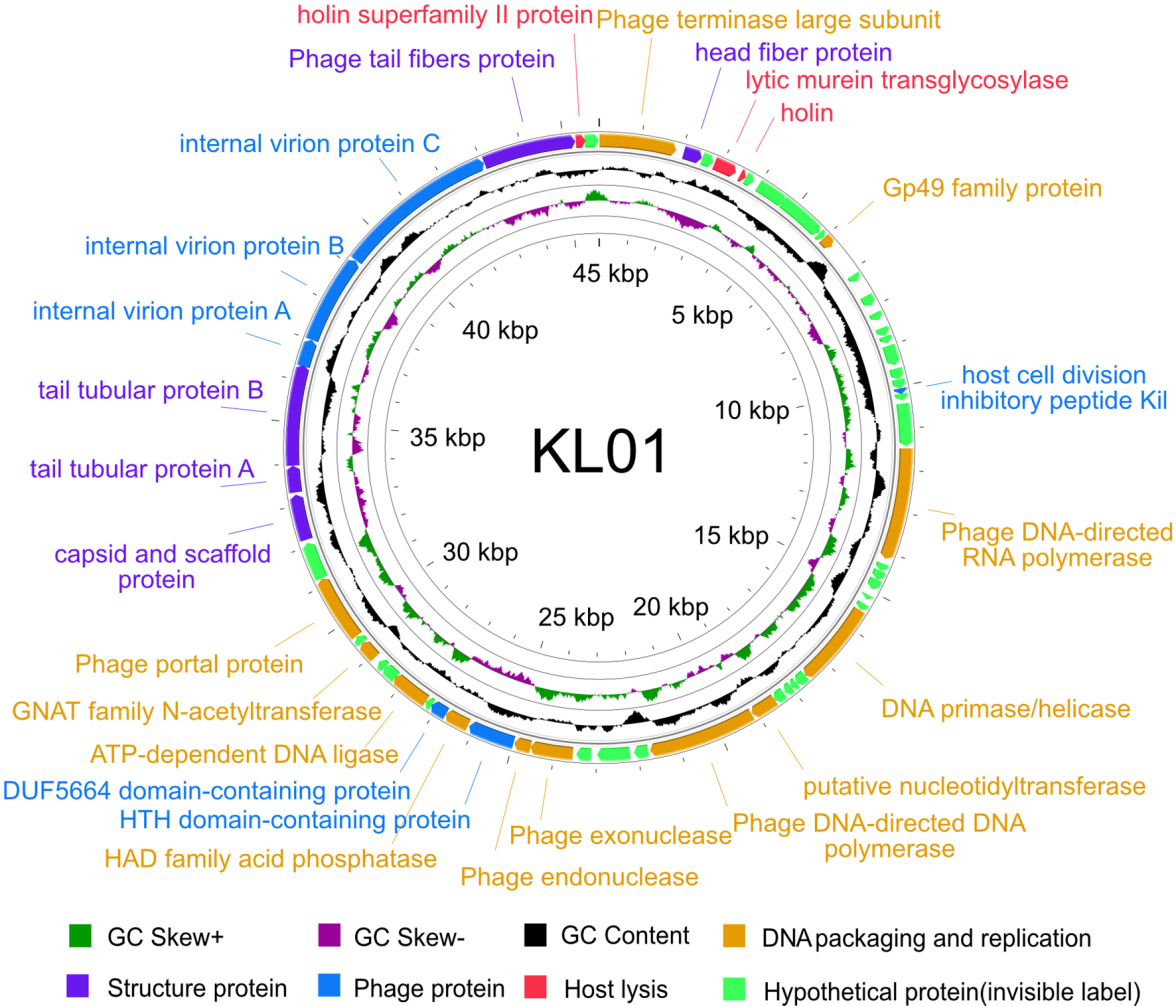
Genome structure of phage KL01. There are four circles in total from the outside to the inside. The outermost circle represents the coded CDS, and red represents the host lysis module, dark green represents the bacteriophage structural protein module, yellow represents the DNA packaging and replication module, light green represents the assumed protein (label hidden), and blue represents the phage protein module; The second black circle shows GC content, which outwardly represents higher than the average GC content of phage, and vice versa, represents lower than the average GC content. The third circle is shown in green and purple, which represent the G + C bias G-C/G + C, respectively. Green indicates that the value is greater than 0, and purple indicates that it is less than 0.

Phage KL01 (GenBank ID: OR591532) was about 45.9 kb of the genome size (Fig. 3) and 42.5% of the GC coverage content, which base distribution was as follows: G = 11,019, 24.03%; C = 8,469, 18.47%; A = 13,558, 29.57%; T = 12,808, 27.93%. KL01 contained 59 ORFs, with a total length of 42,612 bp, accounting for 92.29% of the total length. There was no tRNA identified. In the KL01 genome, 49 ORFs had ATG as the start codon, 7 ORFs had TTG as the start codon, and 3 ORFs had GTG as the start codon. According to the annotation results, 26 ORFs had known functions and 33 ORFs encoded hypothetical proteins. The genome consisted of five main modules, including DNA packaging and replication, host lysis, hypothetical protein, phage protein, and structure protein (Fig. 3 and Table S2).

The KL01 genome contained six structural protein genes, named ORF2, ORF49, ORF51, ORF52, ORF53 and ORF57. The 6 genes encoding structural proteins were very close to each other, with the main capsid protein ORF51 of KL01 being relatively close to phage packaging protein. ORF2, ORF52, ORF53, and ORF57 were related to the coding of phage tail associated proteins, which might play an important role in the process of phage adsorption and infection of host cells. The genes involved in phage DNA replication and regulation were ORF34 (DNA polymerase), ORF22 (RNA polymerase), ORF28 (DNA primer/helicase), ORF33 (nucleotide transferase), ORF39 (endonuclease), and ORF38 (exonuclease). The genes involved in phage structure, packaging, and other processes include ORF1 (large subunit of phage terminating enzyme). ORF4, ORF5, ORF19 and ORF58 encoded genes involved in host bacteria cleavage. Phage KL01 also contained phage virion proteins (ORF54, ORF55, ORF56). It mainly encoded proteins internal virion protein A, internal virion protein B, and internal virion protein C.

### Proteomic features

The total protein was 843 µg, and the concentration was 1.686 µg/µL in 5 mL phage KL01 supernatant. The phage KL01 were identified 18 proteins (Table S2), contains 4 structure proteins (head fiber protein, phage portal protein, capsid and scaffold protein, and tail tubular protein A), 1 lysis-related protein, 8 hypothetical proteins, 2 internal virion proteins, 3 DNA packaging and replication related proteins by Proteomic. The proteins of the lysis function of phage KL01 were lytic murein transglycosylase (ORF4), holin (ORF5), host cell division inhibitory peptide Kil (ORF19) and holin superfamily II protein (ORF58), and the lytic murein transglycosylase was identified by Proteomic. The lytic murein transglycosylase has the largest relative molecular mass and amino acids numbers, which is a stable hydrophilic protein, and the other proteins were stable hydrophobic protein. We found that the holin and holin superfamily II protein had transmembrane region (Fig. S2). Meanwhile, lytic murein transglycosylase and holin had signal peptide, so they were secreted proteins (Fig. S3).

### Comparative genomic and phylogenetic analysis of KL01

**Fig. 4:**
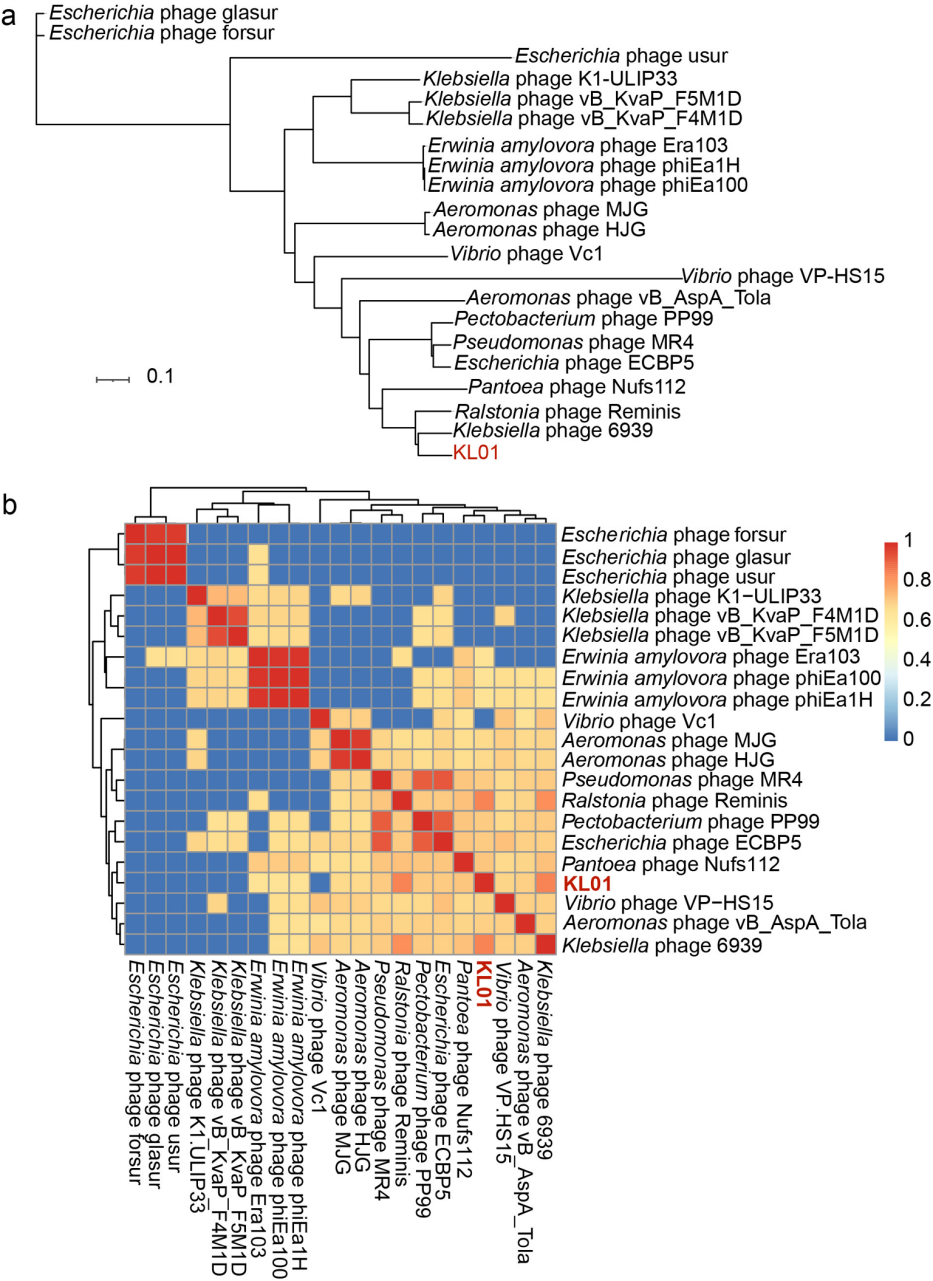
Relationship between phage KL01 and other phages. **a**. Genome-wide evolutionary tree of phage KL01. **b**. Average nucleotide identity (ANI); Full genomes of 21 phages were downloaded from the NCBI database. The value ranges from 0 (0%) to 1 (100%). Blue indicates 0% ANI. Clusters of highly similar phages are highlighted in red and yellow.

Phylogenetic analysis of KL01 genome (Fig. 4a) showed that KL01 is closely related to *Klebsiella* phage 6939 and *Ralstonia* phage Reminis. Meanwhile, the relatively conservative and evolutionarily significant major capsid protein amino acid sequence was also selected to analysis the phylogenetic relationship with other 63 phages (Fig. S4). Phylogenetic analysis of major capsid protein sequences showed that KL01, *Klebsiella* phage 6939 and *Ralstonia* phage Reminis formed a well-supported group, which indicated that they might have originated from the same ancestor.

To determine the genomic similarity between KL01 and other phages, nucleotide sequence blast was performed in NCBI and pairwise ANI was calculated based on the whole genome sequences retrieved from the NCBI database. The KL01 had moderate average nucleotide consistency with *Ralstonia* phage Reminis (GenBank: MN478376.1) and *Klebsiella* phage 6939 (GenBank: OL362271.1), with the identity of 83.09% and 83.04% and the query coverage of 85% and 81%. The ANIb identity analysis showed KL01 to *Klebsiella* phage 6939 and *Ralstonia* phage Reminis are around 80%, which indicating a close relationship between their genomes, and had low similarities with other phages (Fig. 4b). International Committee on the Taxonomy of Viruses (ICTV) has released that the main species demarcation criterion for defining new strain of bacterial, archaeal and viruses is currently set at a genome sequence identity of 95% [49, 50]. Therefore, we preliminarily concluded that the genome of KL01 was significantly different from other phages in the database and predicted that KL01 was a novel species of phage.

**Fig. 5:**
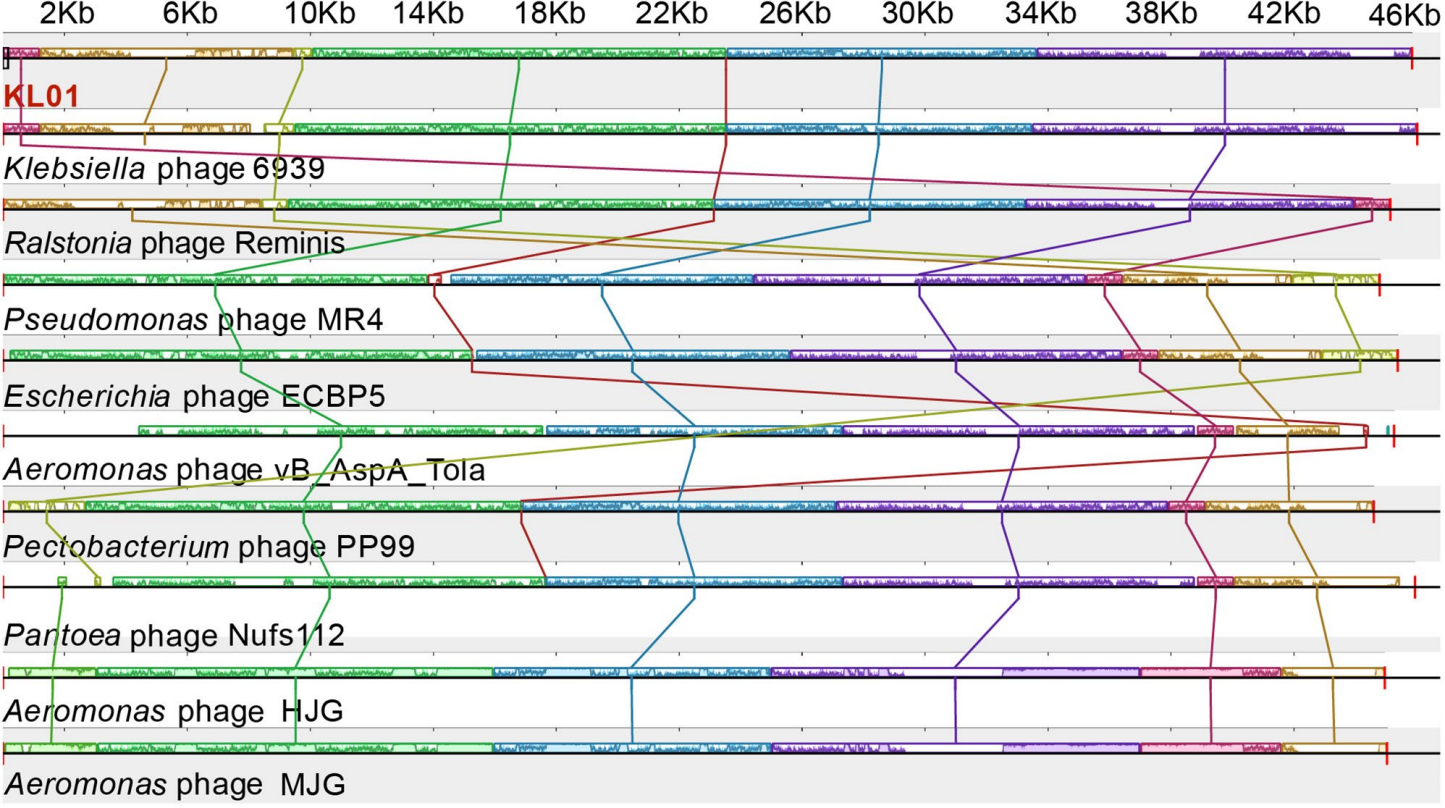
Genomic synteny analysis of KL01. Homologous regions are represented by the same color, the lines indicate that the two gene modules are collinear, and the height of the similar profile within each block corresponds to the average conserved level of the genomic region.

Based on the results of ANI and phage phylogenetic analyses, we selected 9 phages to perform genome collinearity analysis. The results showed that all these genomes contain five large homologous local collinear blocks (LCBs), which are homologous (Fig. 5). In addition, they also contained a small local collinearity region, which is terminase large subunit. KL01 had high collinearity with the genome of the 9 phage strains, and the genome was relatively conserved. There was no large-scale gene rearrangement in different genomes, no replacement or inversion of homologous genes, but there was deletion of large fragments in individual genomes. KL01 had the nearest size and arrangement rules to *Ralstonia* phage Reminis and *Klebsiella* phage 6939 in the homologous region, and had low similar in the homologous region of 2–10 kb and 34–46 kb. Compared with *Aeromonas* phage HJG and *Aeromonas* phage MJG, phage KL01 did not have LCBs that in 0–4 kb, which was unique in *Aeromonas* phage HJG and *Aeromonas* phage MJG.

**Fig. 6:**
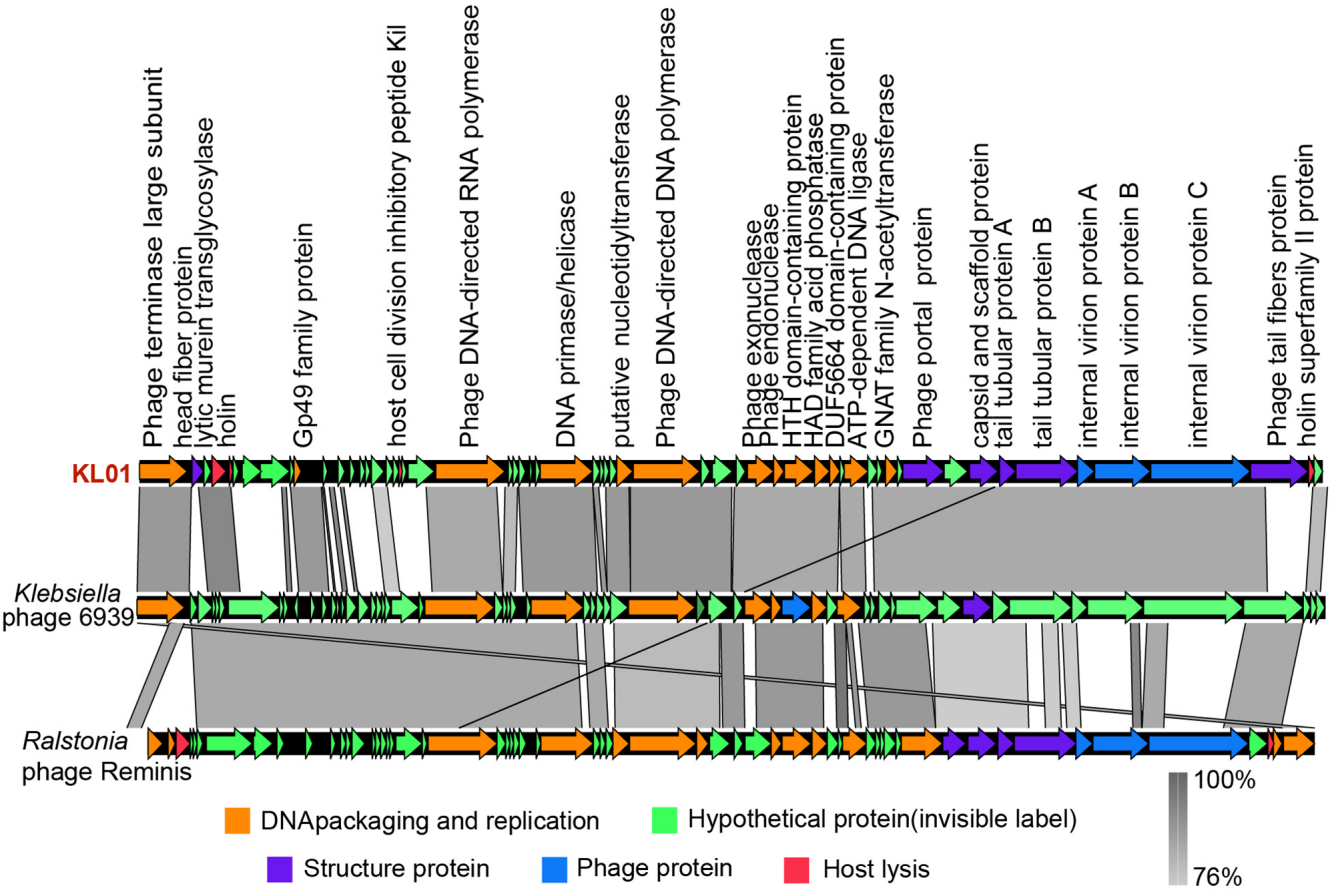
Genome comparison of KL01 and its phage relatives. Genetic comparisons were performed using tBLASTx, and was visualized using Easyfig 2.2. The shaded lines reflect the degree of homology between pairs of phages, and the arrows represent the locations of ORFs. ORFs are colored according to gene function, as indicated at the bottom of the figure.

In addition, KL01 had little hypothetical proteins and had no reverse transcription when compared with *Klebsiella* phage 6939 (Fig. 6). The functional module of KL01 was consistent with the *Ralstonia* phage Reminis, but the size and arrangement of the ORF fragments were inconsistent. In the DNA packaging and replication module, *Ralstonia* phage Reminis has one more terminase large subunit protein than KL01. Meanwhile, between DNA polymerase and RNA polymerase, *Ralstonia* phage Reminis had more hypothetical protein than KL01. For the structural protein module, capsid had strong structural identity, but tail fibers were unique to phage KL01. For the host lysis, phage KL01 contained more holin related proteins. Therefore, phage KL01 had homology with *Ralstonia* Reminis and *Klebsiella* phage 6939, but they are different isolates. KL01 belongs to the *Autographiviridae* within the class *Caudoviricetes*.

## Discussion

The dominant bacterial that we isolated were Proteobacteria and Firmicutes, which was similar to the results of previous study in this area [51]. Notably, we also isolated some *Klebsiella* species including *K. quasipneumoniae* from the study area. *Klebsiella* is common in hospital pathogen, but it had also been found in this karst research area, which might be related to nearby residential areas and sewage treatment plants [52]. Meanwhile, *K. quasipneumoniae* has become the most potentially pathogenic species due to its ability to produce highly virulent clones [53–55]. *K. quasipneumoniae* strains are multidrug-resistant, and prone to acquire and transfer ARGs [56–58]. Therefore, it shows that there are some basic conditions pathogens in this environment, which cause the ecological risks of karst water resources.

The known *Klebsiella* phages from *Podoviridae*, *Siphoviridae*, *Drexlerviridae*, *Myoviridae*, *Autographiviridae*, and *Demerecviridae*, which are mainly derived from hospital wastewater and domestic sewage [59, 60]. The growth curve of phage has important reference value for the application of phage therapy. The short latent period and medium burst size of phage KL01 are comparable to many of the published *Klebsiella* phages [61–63]. The plaque in KL01 is small and pinpoint. The clear halo around the plaque indicates that there is endolysin, which is produced by phage replication in the host [64]. At the same time, halo zone also explained by the presence of depolymerase in the phage genome. The phage encoded depolymerases could specifically cleave polysaccharides (e.g. capsular polysaccharides, exopolysaccharides or lipopolysaccharides) of the host bacteria and therefore provide an important advantage for phages to adsorb onto their hosts [65, 66]. KL01 could infect one of 32 isolated bacteria, which was the host bacteria. Therefore, KL01 can quickly and accurately lysis *K. quasipneumoniae* when applied in the environment, with less impact on the in situ ecology of the environment.

Phage genome sizes vary greatly [67]. Through NCBI research, it is found that the genome size of the known *Klebsiella* phage is generally between ~17 kb and 269 kb, while the genome size of the phage KL01 is 45.9 kb, which is one of the smaller sequenced *Klebsiella* phage genome. The GC content of different phages is also very different, and the difference of GC content can lead to great differences in the protein and amino acid content of phages [68]. KL01 encodes holin, which is an essential membrane protein that participates in host cell lysis. Holin acts as a “protein clock” for bacteriophage infection, regulating the time of the bacteriophage infection cycle and mediating host lysis at a specific time point [61, 69]. Phage KL01 also encodes portal protein, which has been further confirmed in proteomics. Portal proteins are dodecameric assemblies, mainly found in tailed bacteriophages and herpesvirus [70]. The portal protein functions as a bidirectional gateway through which a virus can exchange DNA with the outside environment [71]. During virus morphogenesis, the portal protein recruits small and large terminase subunits that assemble to form a genome-packaging motor [72].

Due to the increase in antibiotic-resistant contamination caused by pathogens such as *Klebsiella pneumoniae*, phage therapy has become a viable alternative to existing antimicrobial chemotherapies [70, 72, 73]. Phage therapy can be used as an alternative to antibiotics because it induces a lethal effect on the host bacteria through lysis [74]. Studies have shown that intraperitoneal injection of phage BL02, which the host bacteria was *K. pneumoniae* can effectively improve the survival rate of mice suffering from bacteremia [75]. The unfavorable characteristics of therapeutic phages generally include carrying virulence and resistance genes [76]. Because phage not only spread resistance genes by horizontal gene transfer, but also regulate the expression of virulence genes by coding [77]. The presence of these two genes will bring non-negligible risks to the practical application of phages in the environment. Therefore, whole-genome sequencing is critical in identifying the unfavorable characteristics of phages [78]. In the case of phage KL01, no resistance genes or toxicity genes were found in its genome, indicating that its use in the environment has little ecological risk and provides a safety guarantee [79]. Phages are useful in monitoring pathogens and biofilm contamination in wastewater treatment systems [80]. Bacteriophages can be used in wastewater to remove pathogenic bacteria and mitigated proliferation of antibiotic resistant strains [81].

## Conclusions

Potentially pathogenic bacteria were detected in the southwest China’s karst water. Meanwhile, a new *K. quasipneumoniae* phage KL01 was isolated with strong lytic ability but without virulence genes and antibiotic resistance genes. These findings suggest that there were some conditionally pathogenic bacteria with certain ecological risk to karst environment. However, phage resources were also explored for application. Phage KL01 warrants further investigation for the biological control in karst environment.

### Electronic supplementary material

Below is the link to the electronic supplementary material.

Supplementary Material 1

## Data Availability

No datasets were generated or analysed during the current study.

## References

[1] Buckerfield SJ, Waldron S, Quilliam RS, Naylor LA, Li S, Oliver DM (2019). How can we improve understanding of faecal indicator dynamics in karst systems under changing climatic, population, and land use stressors?–Research opportunities in SW China. Sci Total Environ.

[2] Jiang Z, Xia R, Shi J, Pei J, Liang HES (2006). The Application effects and Exploitation Capacity of Karst Underground Water resources in Southwest China. J Geosci.

[3] Gutiérrez F, Parise M, De Waele J, Jourde H (2014). A review on natural and human-induced geohazards and impacts in karst. Earth Sci Rev.

[4] Ford D, Williams PD. Karst hydrogeology and geomorphology. Wiley; 2007.

[5] Yuan J, Xu F, Deng G, Tang Y, Li P (2017). Hydrogeochemistry of shallow groundwater in a karst aquifer system of Bijie City,Guizhou Province. Water.

[6] Chen L, Lang H, Liu F, Jin S, Yan T (2018). Presence of antibiotics in shallow groundwater in the northern and southwestern regions of China. Groundwater.

[7] Xiang S, Wang X, Ma W, Liu X, Zhang B, Huang F, Liu F, Guan X (2020). Response of microbial communities of karst river water to antibiotics and microbial source tracking for antibiotics. Sci Total Environ.

[8] Liu C-l, Wang X-y, Lu D-y. Zhao Y-w: Risk assessment and control countermeasures of southern China’s karst groundwater areal source pollution. Acta Geoscientica Sinica 2017:910–8.

[9] Ashbolt NJ (2004). Microbial contamination of drinking water and disease outcomes in developing regions. Toxicology.

[10] Nguyen HTM, Le QTP, Garnier J, Janeau J-L, Rochelle-Newall E (2016). Seasonal variability of faecal indicator bacteria numbers and die-off rates in the Red River basin, North Viet Nam. Sci Rep.

[11] Kaiser RA, Polk JS, Datta T, Parekh RR, Agga GE (2022). Occurrence of antibiotic resistant bacteria in urban karst groundwater systems. Water.

[12] Sinreich M, Pronk M, Kozel R (2014). Microbiological monitoring and classification of karst springs. Environ Earth Sci.

[13] Brad T, Fekete A, Şandor MS, Purcărea C (2015). Natural attenuation potential of selected hydrokarst systems in the Carpathian Mountains (Romania). Water Sci Technology: Water Supply.

[14] Dedrick RM, Guerrero-Bustamante CA, Garlena RA, Russell DA, Ford K, Harris K, Gilmour KC, Soothill J, Jacobs-Sera D, Schooley RT (2019). Engineered bacteriophages for treatment of a patient with a disseminated drug-resistant Mycobacterium abscessus. Nat Med.

[15] Schooley RT, Biswas B, Gill JJ, Hernandez-Morales A, Lancaster J, Lessor L, Barr JJ, Reed SL, Rohwer F, Benler S (2017). Development and use of personalized bacteriophage-based therapeutic cocktails to treat a patient with a disseminated resistant Acinetobacter baumannii infection. Antimicrob Agents Chemother.

[16] D’Accolti M, Soffritti I, Mazzacane S, Caselli E (2021). Bacteriophages as a potential 360-degree pathogen control strategy. Microorganisms.

[17] Wen C, Ai C, Lu S, Yang Q, Liao H, Zhou S (2022). Isolation and characterization of the lytic Pseudoxanthomonas kaohsiungensi phage PW916. Viruses.

[18] Ye M, Sun M, Huang D, Zhang Z, Zhang H, Zhang S, Hu F, Jiang X, Jiao W (2019). A review of bacteriophage therapy for pathogenic bacteria inactivation in the soil environment. Environ Int.

[19] Pallavali R, Shin D, Choi J (2023). Phage-based Biocontrol of antibiotic-resistant bacterium isolated from Livestock Wastewater Treatment Plant. Water.

[20] Wang X, Wei Z, Yang K, Wang J, Jousset A, Xu Y, Shen Q, Friman V-P (2019). Phage combination therapies for bacterial wilt disease in tomato. Nat Biotechnol.

[21] Stolp H, Starr MP. Principles of isolation, cultivation, and conservation of bacteria. The Prokaryotes: A Handbook on Habitats, Isolation, and Identification of Bacteria 1981:135–175.

[22] Sinha M, Kapley A, Purohit HJ (2008). Study of biodiversity of Klebsiella Sp. World J Microbiol Biotechnol.

[23] Kim O-S, Cho Y-J, Lee K, Yoon S-H, Kim M, Na H, Park S-C, Jeon YS, Lee J-H, Yi H (2012). Introducing EzTaxon-e: a prokaryotic 16S rRNA gene sequence database with phylotypes that represent uncultured species. Int J Syst Evol MicroBiol.

[24] Kumar S, Stecher G, Li M, Knyaz C (2018). MEGA X: molecular evolutionary genetics analysis across computing platforms. Mol Biol Evol.

[25] Dalğıç D, Kandemir T, Üçkayabaşı A, Nağıyev T (2023). Relationship of Hypervirulent capsular genotypes of Klebsiella pneumoniae with antibiotic susceptibility and Beta-lactamase genes. Mikrobiyoloji Bulteni.

[26] Khare V, Gupta P, Haider F, Begum R (2017). Study on MICs of tigecycline in clinical isolates of carbapenem resistant Enterobacteriaceae (CRE) at a tertiary care centre in North India. J Clin Diagn Research: JCDR.

[27] Carlson K. Working with bacteriophages: common techniques and methodological approaches. CRC press Boca Raton, FL; 2005.

[28] Van Twest R, Kropinski AM. Bacteriophage enrichment from water and soil. Bacteriophages: Methods and Protocols, Volume 1: Isolation, Characterization, and Interactions 2009:15–21. 10.1007/978-1-60327-164-6_2 19066806

[29] Sambrook J, Fritsch EF, Maniatis T. Molecular cloning: a laboratory manual. Cold Spring Harbor Laboratory Press; 1989.

[30] Clokie MR, Kropinski A. Methods and protocols, 1: isolation, characterization, and interactions. Methods Mol Biology Humana Press 2009:69–81.

[31] Tu AVT, Pham-Khanh NH, Nguyen SH, Sunahara H, Xuan TDT, Kamei K (2023). Isolation, characterization, and complete genome sequence of vibrio phage KIT04, a novel lytic phage of the subfamily ermolyevavirinae. Virology.

[32] Passaretti P, Khan I, Dafforn TR, Goldberg Oppenheimer P (2020). Improvements in the production of purified M13 bacteriophage bio-nanoparticle. Sci Rep.

[33] Kreienbaum M, Dörrich AK, Brandt D, Schmid NE, Leonhard T, Hager F, Brenzinger S, Hahn J, Glatter T, Ruwe M (2020). Isolation and characterization of Shewanella phage thanatos infecting and lysing Shewanella oneidensis and promoting nascent biofilm formation. Front Microbiol.

[34] Li F, Li L, Zhang Y, Bai S, Sun L, Guan J, Zhang W, Cui X, Feng J, Tong Y (2022). Isolation and characterization of the novel bacteriophage vB_SmaS_BUCT626 against Stenotrophomonas maltophilia. Virus Genes.

[35] Abedon ST (2016). Phage therapy dosing: the problem (s) with multiplicity of infection (MOI). Bacteriophage.

[36] Benala M, Vaiyapuri M, Visnuvinayagam S, George JC, Raveendran K, George I, Mothadaka MP, Badireddy MR (2021). A revisited two-step microtiter plate assay: optimization of in vitro multiplicity of infection (MOI) for coliphage and Vibriophage. J Virol Methods.

[37] Yin Y, Liu D, Yang S, Almeida A, Guo Q, Zhang Z, Deng L, Wang D (2019). Bacteriophage potential against Vibrio parahaemolyticus biofilms. Food Control.

[38] Tokman JI, Kent DJ, Wiedmann M, Denes T (2016). Temperature significantly affects the plaquing and adsorption efficiencies of Listeria phages. Front Microbiol.

[39] Kutter E. Phage host range and efficiency of plating. Bacteriophages: Methods and protocols, Volume 1: Isolation, characterization, and interactions 2009:141–149. 10.1007/978-1-60327-164-6_14 19066818

[40] Joshi N, Fass J. Sickle: A sliding-window, adaptive, quality-based trimming tool for FastQ files (Version 1.33)[Software]. 2011.

[41] Bankevich A, Nurk S, Antipov D, Gurevich AA, Dvorkin M, Kulikov AS, Lesin VM, Nikolenko SI, Pham S, Prjibelski AD (2012). SPAdes: a new genome assembly algorithm and its applications to single-cell sequencing. J Comput Biol.

[42] Parks DH, Imelfort M, Skennerton CT, Hugenholtz P, Tyson GW (2015). CheckM: assessing the quality of microbial genomes recovered from isolates, single cells, and metagenomes. Genome Res.

[43] Langmead B, Salzberg SL (2012). Fast gapped-read alignment with Bowtie 2. Nat Methods.

[44] Besemer J, Lomsadze A, Borodovsky M (2001). GeneMarkS: a self-training method for prediction of gene starts in microbial genomes. Implications for finding sequence motifs in regulatory regions. Nucleic Acids Res.

[45] Stothard P, Grant JR, Van Domselaar G (2019). Visualizing and comparing circular genomes using the CGView family of tools. Brief Bioinform.

[46] Pritchard L, Glover RH, Humphris S, Elphinstone JG, Toth IK (2016). Genomics and taxonomy in diagnostics for food security: soft-rotting enterobacterial plant pathogens. Anal Methods.

[47] Larkin MA, Blackshields G, Brown NP, Chenna R, McGettigan PA, McWilliam H, Valentin F, Wallace IM, Wilm A, Lopez R. Clustal W and Clustal X version 2.0. bioinformatics 2007, 23:2947–2948. 10.1093/bioinformatics/btm404 17846036

[48] Darling AC, Mau B, Blattner FR, Perna NT (2004). Mauve: multiple alignment of conserved genomic sequence with rearrangements. Genome Res.

[49] Adriaenssens EM, Brister JR (2017). How to name and classify your phage: an informal guide. Viruses.

[50] Zeng H, He W, Li C, Zhang J, Ling N, Ding Y, Xue L, Chen M, Wu H, Wu Q (2019). Complete genome analysis of a novel phage GW1 lysing Cronobacter. Arch Virol.

[51] Zhang B, Qin S, Guan X, Jiang K, Jiang M, Liu F (2021). Distribution of antibiotic resistance genes in Karst River and its ecological risk. Water Res.

[52] Zurabov F, Zhilenkov E (2021). Characterization of four virulent Klebsiella pneumoniae bacteriophages, and evaluation of their potential use in complex phage preparation. Virol J.

[53] Holt KE, Wertheim H, Zadoks RN, Baker S, Whitehouse CA, Dance D, Jenney A, Connor TR, Hsu LY, Severin J. Genomic analysis of diversity, population structure, virulence, and antimicrobial resistance in Klebsiella pneumoniae, an urgent threat to public health. Proceedings of the National Academy of Sciences 2015, 112:E3574-E3581. 10.1073/pnas.1501049112 PMC4500264 26100894

[54] Bengoechea JA, Sa Pessoa J (2019). Klebsiella pneumoniae infection biology: living to counteract host defences. FEMS Microbiol Rev.

[55] Choby J, Howard-Anderson J, Weiss D (2020). Hypervirulent Klebsiella pneumoniae–clinical and molecular perspectives. J Intern Med.

[56] Navon-Venezia S, Kondratyeva K, Carattoli A (2017). Klebsiella pneumoniae: a major worldwide source and shuttle for antibiotic resistance. FEMS Microbiol Rev.

[57] Shankar C, Nabarro LE, Anandan S, Ravi R, Babu P, Munusamy E, Jeyaseelan V, Rupali P, Verghese VP, Veeraraghavan B (2018). Extremely high mortality rates in patients with carbapenem-resistant, hypermucoviscous Klebsiella pneumoniae blood stream infections. J Assoc Physicians India.

[58] Tacconelli E, Carrara E, Savoldi A, Harbarth S, Mendelson M, Monnet DL (2018). Discovery, research, and development of new antibiotics: the WHO priority list of antibiotic-resistant bacteria and tuberculosis. Lancet Infect Dis.

[59] Townsend EM, Kelly L, Gannon L, Muscatt G, Dunstan R, Michniewski S, Sapkota H, Kiljunen SJ, Kolsi A, Skurnik M (2021). Isolation and characterization of Klebsiella phages for phage therapy. Therapy Appl Res.

[60] Li F, Tian F, Nazir A, Sui S, Li M, Cheng D, Nong S, Ali A, KaKar M-U, Li L (2022). Isolation and genomic characterization of a novel Autographiviridae bacteriophage IME184 with lytic activity against Klebsiella pneumoniae. Virus Res.

[61] Lu N, Sun Y, Wang Q, Qiu Y, Chen Z, Wen Y, Wang S, Song Y (2020). Cloning and characterization of endolysin and holin from Streptomyces avermitilis bacteriophage phiSASD1 as potential novel antibiotic candidates. Int J Biol Macromol.

[62] Morozova V, Kozlova Y, Jdeed G, Tikunov A, Ushakova T, Bardasheva A, Zhirakovskaia E, Poletaeva Y, Ryabchikova E, Tikunova NV (2022). A Novel Aeromonas popoffii Phage AerP_220 proposed to be a Member of a New Tolavirus Genus in the Autographiviridae Family. Viruses.

[63] Pu M, Han P, Zhang G, Liu Y, Li Y, Li F, Li M, An X, Song L, Chen Y (2022). Characterization and Comparative Genomics Analysis of a New Bacteriophage BUCT610 against Klebsiella pneumoniae and Efficacy Assessment in Galleria mellonella Larvae. Int J Mol Sci.

[64] Yuan Y, Li X, Wang L, Li G, Cong C, Li R, Cui H, Murtaza B, Xu Y (2021). The endolysin of the Acinetobacter baumannii phage vB_AbaP_D2 shows broad antibacterial activity. Microb Biotechnol.

[65] Schmelcher M, Loessner MJ (2016). Bacteriophage endolysins: applications for food safety. Curr Opin Biotechnol.

[66] Knecht LE, Veljkovic M, Fieseler L (2020). Diversity and function of phage encoded depolymerases. Front Microbiol.

[67] Dion MB, Oechslin F, Moineau S (2020). Phage diversity, genomics and phylogeny. Nat Rev Microbiol.

[68] Olo Ndela E, Roux S, Henke C, Sczyrba A, Sime Ngando T, Varsani A, Enault F (2023). Reekeekee-and roodoodooviruses, two different Microviridae clades constituted by the smallest DNA phages. Virus Evol.

[69] Cahill J, Young R (2019). Phage lysis: multiple genes for multiple barriers. Adv Virus Res.

[70] Law N, Aslam S (2020). Phage therapy: primer and role in the treatment of MDROs. Curr Infect Dis Rep.

[71] Hou C-FD, Swanson NA, Li F, Yang R, Lokareddy RK, Cingolani G (2022). Cryo-EM structure of a kinetically trapped dodecameric portal protein from the Pseudomonas-phage PaP3. J Mol Biol.

[72] Luong T, Salabarria A-C, Roach DR (2020). Phage therapy in the resistance era: where do we stand and where are we going?. Clin Ther.

[73] Loh B, Gondil VS, Manohar P, Khan FM, Yang H, Leptihn S (2021). Encapsulation and delivery of therapeutic phages. Appl Environ Microbiol.

[74] Dufour N, Debarbieux L (2017). Phage therapy: a realistic weapon against multidrug resistant bacteria. Med Sciences: M/S.

[75] Liang Z, Shi Y-L, Peng Y, Xu C, Zhang C, Chen Y, Luo X-Q, Li Q-M, Zhao C-L, Lei J (2023). BL02, a phage against carbapenem-and polymyxin-B resistant Klebsiella pneumoniae, isolated from sewage: a preclinical study. Virus Res.

[76] Fernández L, Gutiérrez D, García P, Rodríguez A (2019). The perfect bacteriophage for therapeutic applications—a quick guide. Antibiotics.

[77] Feng J, Gao L, Li L, Zhang Z, Wu C, Li F, Tong Y (2021). Characterization and genome analysis of novel Klebsiella phage BUCT556A with lytic activity against carbapenemase-producing Klebsiella pneumoniae. Virus Res.

[78] Casey E, Van Sinderen D, Mahony J (2018). In vitro characteristics of phages to guide ‘real life’ phage therapy suitability. Viruses.

[79] Mulani MS, Kumkar SN, Pardesi KR (2022). Characterization of Novel Klebsiella Phage PG14 and its Antibiofilm Efficacy. Microbiol Spectr.

[80] Ji M, Liu Z, Sun K, Li Z, Fan X, Li Q (2021). Bacteriophages in water pollution control: advantages and limitations. Front Environ Sci Eng.

[81] Mathieu J, Yu P, Zuo P, Da Silva ML, Alvarez PJ (2019). Going viral: emerging opportunities for phage-based bacterial control in water treatment and reuse. Acc Chem Res.

