# Supplementary material for: Bacterial isolation and genome analysis of a novel Klebsiella quasipneumoniae phage in southwest China’s karst area
Source: Virol J. 2024 Mar 6;21:56. doi: 10.1186/s12985-024-02321-1 (PMC10916049; doi:10.1186/s12985-024-02321-1)
Supplement: Supplementary file 1 — Supplementary Material 1 [file 12985_2024_2321_MOESM1_ESM.docx]

### Supplemental materials

### Table S1. Host Range Analysis Results of Bacteriophage KL01

| Bacteria | KL01 | Bacteria | KL01 |
| --- | --- | --- | --- |
| *Bacillus drentensis* | — | *Klebsiella aerogenes* | — |
| *Bacillus cereus* | — | *Kurthia gibsonii* | — |
| *Bacillus thuringiensis* | — | *Microbacterium esteraromaticum* | — |
| *Citrobacter freundii* | — | *Microbacterium oxydans* | — |
| *Comamonas aquatica* | — | *Pseudomonas* sp. | — |
| *Enterococcus* sp. | — | *Pseudomonas fluorescens* | — |
| *Enterococcus faecalis* | — | *Paenibacillus* sp. | — |
| *Enterobacter hormaechei* | — | *Paenibacillus jamilae* | — |
| *Enterococcus faecium* | — | *Paenibacillus polymyxa* | — |
| *Exiguobacterium* | — | *Rhodococcus* sp. | — |
| *Escherichia coli* | — | *Rhodococcus erythropolis* | — |
| *Klebsiella quasipneumoniae* | + | *Rummeliibacillus stabekisii* | — |
| *Klebsiella oxytoca* | — | *Serratia fonticola* | — |
| *Klebsiella pneumoniae* | — | *Sporosarcina saromensis* | — |

### Table S2. Functional Annotation of Phage KL01 Gene

| ORF | Start | End | Strand | Length | Start codon | Peptides | Unique  Peptides | Possible function | Query coverage | Identity |
| --- | --- | --- | --- | --- | --- | --- | --- | --- | --- | --- |
| 1 | 7 | 1854 | → | 1848 | TTG |  |  | Phage terminase large subunit | 97 | 100 |
| 2 | 2039 | 2488 | → | 450 | ATG | 4 | 4 | head fiber protein | 100 | 100 |
| 3 | 2502 | 2780 | → | 279 | ATG |  |  | hypothetical protein | 100 | 100 |
| 4 | 2815 | 3366 | → | 552 | ATG | 8 | 8 | lytic murein transglycosylase | 100 | 100 |
| 5 | 3484 | 3633 | → | 150 | ATG |  |  | holin | 100 | 100 |
| 6 | 3633 | 3845 | → | 213 | ATG |  |  | hypothetical protein | 100 | 95.70 |
| 7 | 3983 | 4732 | → | 750 | TTG |  |  | hypothetical protein | 96 | 62.30 |
| 8 | 4686 | 5819 | → | 1134 | ATG |  |  | hypothetical protein | 97 | 100 |
| 9 | 5830 | 5985 | → | 156 | ATG |  |  | hypothetical protein | 100 | 96.08 |
| 10 | 5987 | 6265 | → | 279 | TTG | 4 | 4 | Gp49 family protein | 100 | 100 |
| 11 | 7109 | 7291 | → | 183 | ATG |  |  | hypothetical protein | 100 | 91.67 |
| 12 | 7693 | 7959 | → | 267 | ATG |  |  | hypothetical protein | 100 | 100 |
| 13 | 8150 | 8344 | → | 195 | ATG |  |  | hypothetical protein | 98 | 92.06 |
| 14 | 8541 | 8744 | → | 204 | ATG |  |  | hypothetical protein | 100 | 83.58 |
| 15 | 8756 | 8932 | → | 177 | ATG |  |  | hypothetical protein | 100 | 96.55 |
| 16 | 8992 | 9480 | → | 489 | ATG |  |  | hypothetical protein | 100 | 88.27 |
| 17 | 9562 | 9828 | → | 267 | ATG |  |  | hypothetical protein | 75 | 90.91 |
| 18 | 9828 | 10049 | → | 222 | ATG |  |  | hypothetical protein | 100 | 53.42 |
| 19 | 10046 | 10186 | → | 141 | ATG |  |  | host cell division inhibitory peptide Kil | 100 | 100 |
| 20 | 10173 | 10343 | → | 171 | ATG |  |  | hypothetical protein | 100 | 52.54 |
| 21 | 10412 | 11431 | → | 1020 | ATG | 13 | 13 | hypothetical protein | 100 | 82.35 |
| 22 | 11482 | 14166 | → | 2685 | ATG |  |  | Phage DNA-directed RNA polymerase | 100 | 90.38 |
| 23 | 14293 | 14472 | → | 180 | TTG |  |  | hypothetical protein | 74 | 72.73 |
| 24 | 14472 | 14696 | → | 225 | ATG |  |  | hypothetical protein | 98 | 86.30 |
| 25 | 14696 | 14956 | → | 261 | ATG |  |  | hypothetical protein | 100 | 67.05 |
| 26 | 15115 | 15228 | → | 114 | ATG |  |  | hypothetical protein | 100 | 100 |
| 27 | 15347 | 15520 | → | 174 | ATG |  |  | hypothetical protein | 100 | 80.70 |
| 28 | 15531 | 17561 | → | 2031 | ATG |  |  | DNA primase/helicase | 100 | 96.01 |
| 29 | 17561 | 17839 | → | 279 | ATG | 1 | 1 | hypothetical protein | 100 | 91.30 |
| 30 | 17836 | 18000 | → | 165 | ATG |  |  | hypothetical protein | 100 | 100 |
| 31 | 17993 | 18193 | → | 201 | ATG | 1 | 1 | hypothetical protein | 100 | 87.88 |
| 32 | 18221 | 18496 | → | 276 | ATG |  |  | hypothetical protein | 100 | 98.90 |
| 33 | 18489 | 19121 | → | 633 | ATG |  |  | putative nucleotidyltransferase | 100 | 74.88 |
| 34 | 19134 | 21716 | → | 2583 | ATG | 6 | 6 | Phage DNA-directed DNA polymerase | 100 | 96.74 |
| 35 | 21759 | 22103 | → | 345 | ATG |  |  | hypothetical protein | 100 | 51.75 |
| 36 | 22192 | 22980 | → | 789 | ATG | 15 | 15 | hypothetical protein | 100 | 95.42 |
| 37 | 23124 | 23474 | → | 351 | ATG | 18 | 18 | hypothetical protein | 100 | 88.79 |
| 38 | 23556 | 24578 | → | 1023 | ATG |  |  | Phage exonuclease | 100 | 91.76 |
| 39 | 24575 | 24973 | → | 399 | ATG |  |  | Phage endonuclease | 100 | 95.45 |
| 40 | 24991 | 26118 | → | 1128 | TTG | 10 | 10 | HTH domain-containing protein | 99 | 69.17 |
| 41 | 26151 | 26744 | → | 594 | GTG |  |  | HAD family acid phosphatase | 100 | 100 |
| 42 | 26741 | 27151 | → | 411 | ATG |  |  | DUF5664 domain-containing protein | 71 | 63.92 |
| 43 | 27141 | 27311 | → | 171 | ATG | 1 | 1 | hypothetical protein | 100 | 80.36 |
| 44 | 27308 | 28243 | → | 936 | ATG |  |  | ATP-dependent DNA ligase | 100 | 89.71 |
| 45 | 28218 | 28595 | → | 378 | ATG |  |  | hypothetical protein | 100 | 69.84 |
| 46 | 28579 | 28773 | → | 195 | ATG |  |  | hypothetical protein | 100 | 89.06 |
| 47 | 28906 | 29370 | → | 465 | ATG |  |  | GNAT family N-acetyltransferase | 100 | 100 |
| 48 | 29370 | 29567 | → | 198 | ATG | 1 | 1 | hypothetical protein | 100 | 95.38 |
| 49 | 29577 | 31178 | → | 1602 | ATG | 2 | 2 | Phage portal protein | 100 | 91.56 |
| 50 | 31184 | 32092 | → | 909 | ATG | 11 | 11 | hypothetical protein | 100 | 78.03 |
| 51 | 32170 | 33273 | → | 1104 | ATG | 16 | 16 | capsid and scaffold protein | 100 | 94.01 |
| 52 | 33324 | 33965 | → | 642 | ATG | 2 | 2 | tail tubular protein A | 100 | 95.31 |
| 53 | 33965 | 36379 | → | 2415 | ATG |  |  | tail tubular protein B | 100 | 92.29 |
| 54 | 36345 | 37007 | → | 663 | ATG |  |  | internal virion protein A | 100 | 92.73 |
| 55 | 37008 | 39170 | → | 2163 | GTG | 5 | 5 | internal virion protein B | 100 | 92.50 |
| 56 | 39180 | 43043 | → | 3864 | ATG | 3 | 3 | internal virion protein C | 100 | 94.41 |
| 57 | 43053 | 45287 | → | 2235 | TTG |  |  | Phage tail fibers protein | 100 | 100 |
| 58 | 45297 | 45533 | → | 237 | TTG |  |  | holin superfamily II protein | 97 | 88.16 |
| 59 | 45508 | 45849 | → | 342 | GTG |  |  | hypothetical protein | 100 | 87.61 |


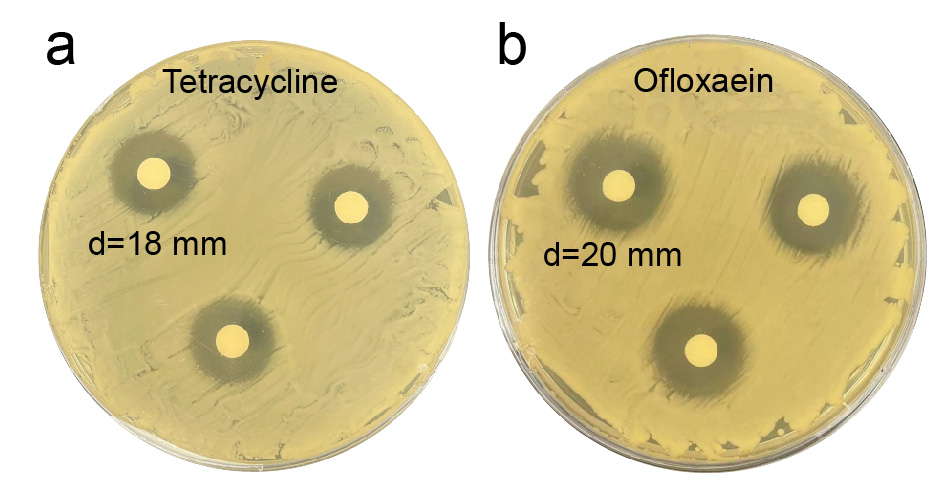


**Fig S1.** **Antibiotic Resistance of the Host Bacteria *Klebsiella Quadrateumoniae*.** Antibiotic resistance of the host bacteria *Klebsiella quadrateumoniae* Antibacterial circle diameter (mm) Ofloxacin tetracycline.


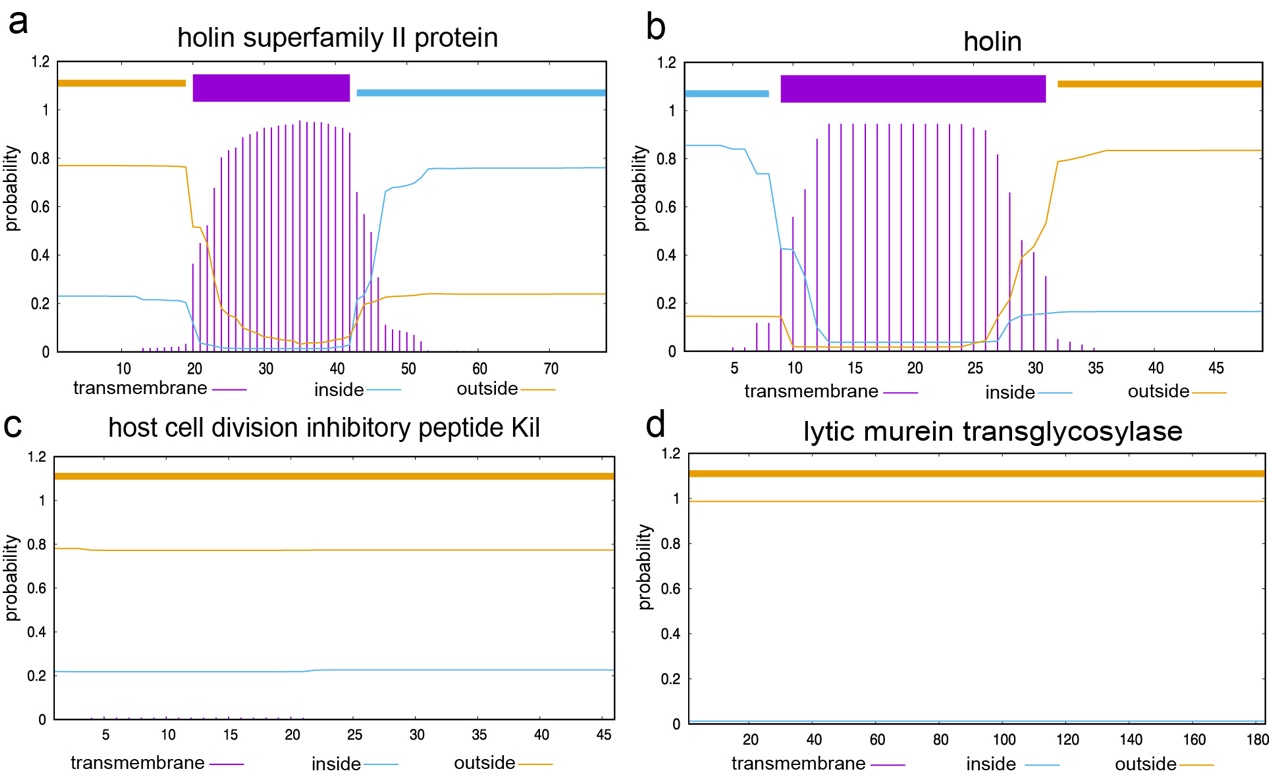


**Fig S2.** **Transmembrane Protein Predictions of Phage KL01 Lysis Protein.** (a) Holin superfamily II protein. (b) Holin. (c) Host cell division inhibitory peptide Kil. (d) Hytic murein transglycosylase.


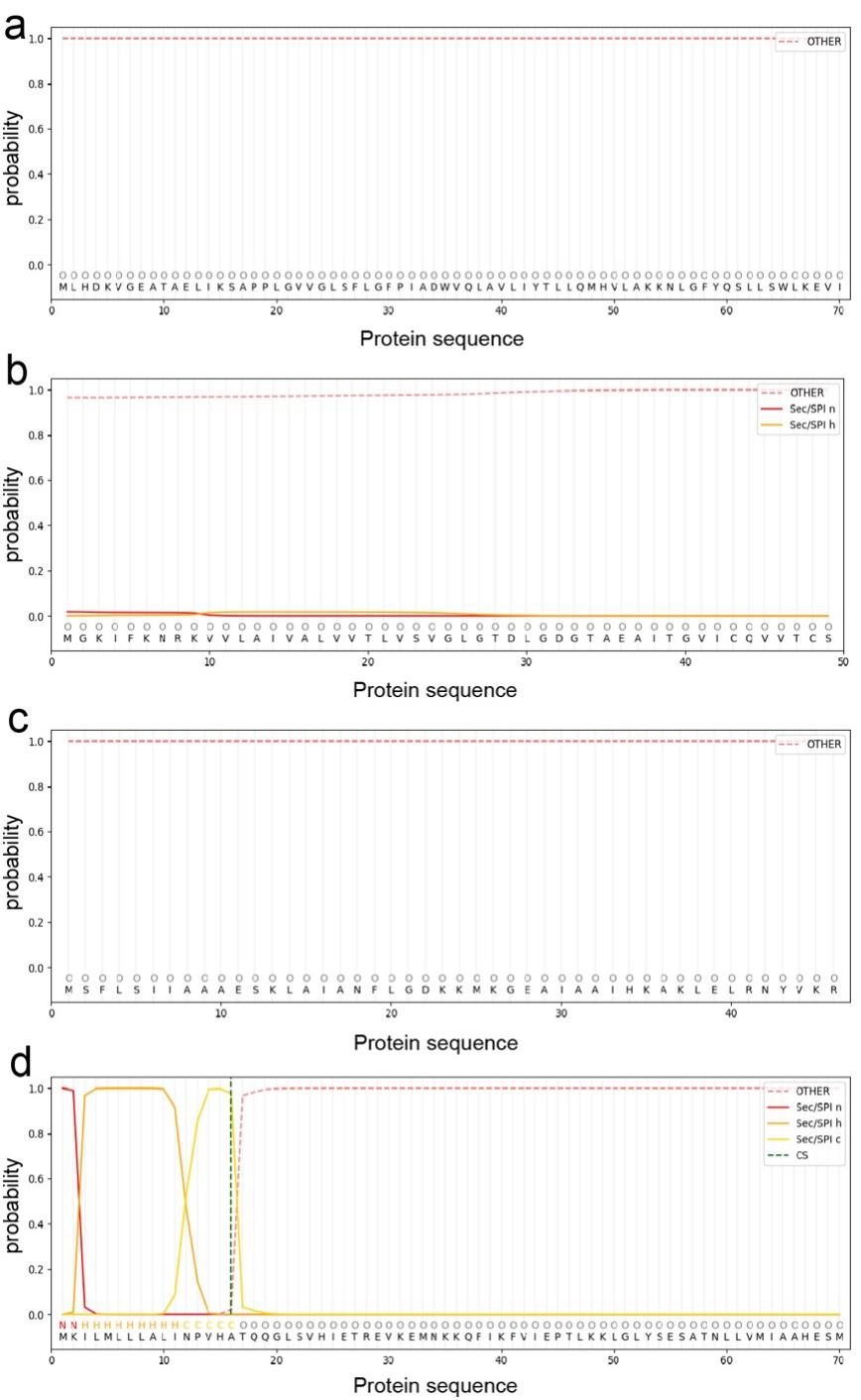


**Fig S3. Signal Peptide Predictions of Phage Kl01 Lysis Protein.** (a) Holin superfamily II protein. (b) Holin. (c) Host cell division inhibitory peptide Kil. (d) Lytic murein transglycosylase.


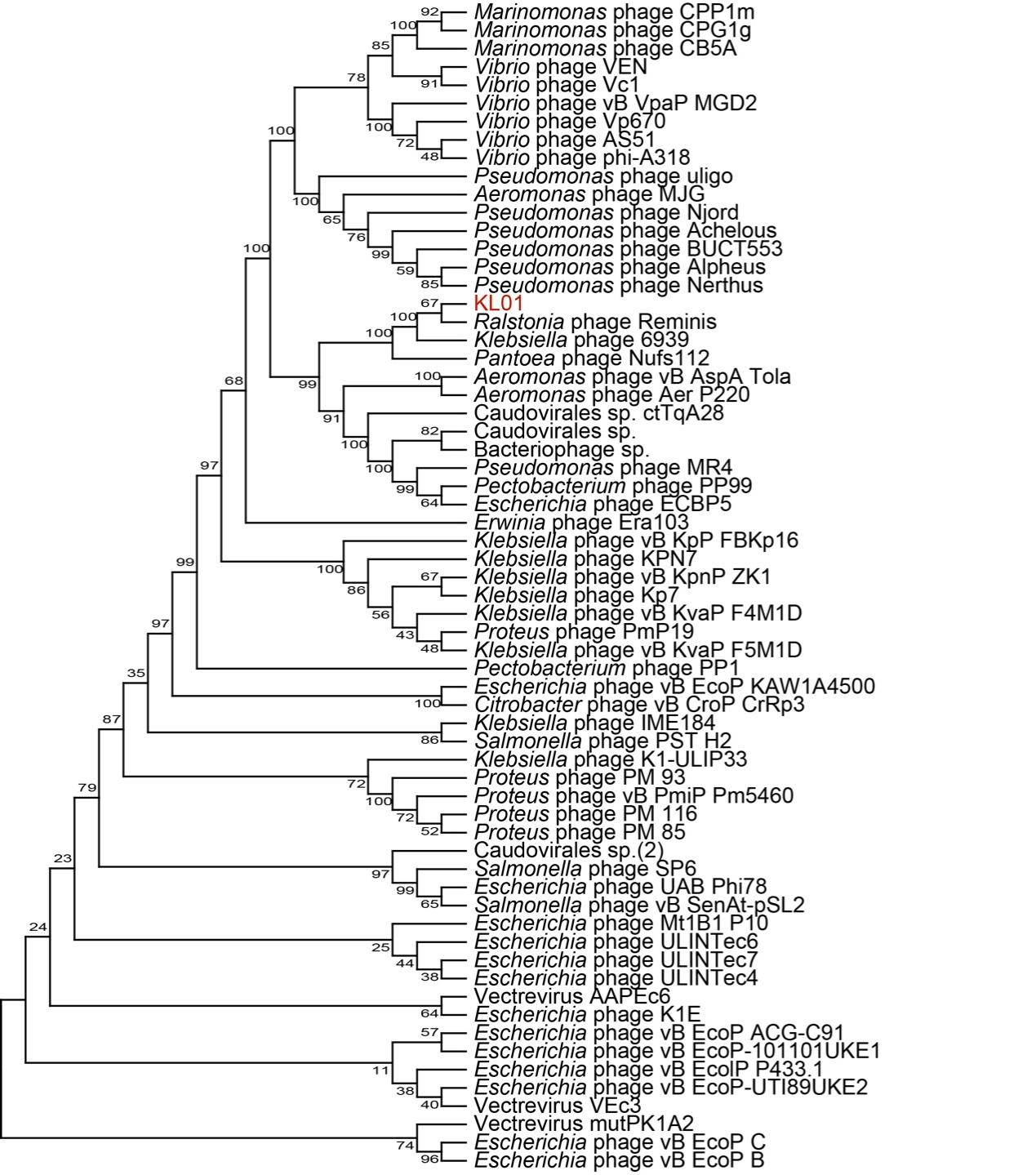


**Fig S4. Phylogenetic Tree of Capsid Protein of Bacteriophage KL01**
